# Vitamin D and Chronic Kidney Disease Association with Mineral and Bone Disorder: An Appraisal of Tangled Guidelines

**DOI:** 10.3390/nu15071576

**Published:** 2023-03-24

**Authors:** Jordi Bover, Elisabet Massó, Laia Gifre, Carlo Alfieri, Jordi Soler-Majoral, Maria Fusaro, Jordi Calabia, Rosely Rodríguez-Pena, Néstor Rodríguez-Chitiva, Víctor López-Báez, Maya Sánchez-Baya, Iara da Silva, Armando Aguilar, Misael C. Bustos, Natacha Rodrigues, Jonathan S. Chávez-Iñiguez, Gregorio Romero-González, José Manuel Valdivielso, Pablo Molina, José L. Górriz

**Affiliations:** 1 Department of Nephrology, University Hospital Germans Trias i Pujol, 08916 Badalona, Spain; 2 REMAR-IGTP Group, Research Institute Germans Trias i Pujol, Can Ruti Campus, 08916 Badalona, Spain; 3 Rheumatology Service, University Hospital Germans Trias i Pujol, 08916 Badalona, Spain; 4 Nephrology, Dialysis and Renal Transplantation, Fondazione IRCCS Ca’Granda Ospedale Maggiore Policlinico, 20122 Milan, Italy; 5 Department of Clinical Sciences and Community Health, University of Milan, 20122 Milan, Italy; 6 National Research Council (CNR), 56124 Pisa, Italy; 7 Department of Medicine, University of Padua, 35128 Padova, Italy; 8 Department of Nephrology, University Hospital Josep Trueta, 17007 Girona, Spain; 9 Department of Nephrology, Instituto Mexicano del Seguro Social, Hospital General de Zona No. 2, Tuxtla Gutiérrez 29000, Mexico; 10 Department of Nephrology, Pontificia Catholic University of Chile, Santiago 8331150, Chile; 11 Division of Nephrology and Renal Transplantation, Department of Medicine, Centro Hospitalar Universitário Lisboa Norte, EPE, 1649-028 Lisboa, Portugal; 12 Department of Nephrology, Hospital Civil de Guadalajara Fray Antonio Alcalde, Guadalajara 44280, Mexico; 13 Centro Universitario de Ciencias de la Salud CUCS, Guadalajara University, Guadalajara 44340, Mexico; 14 Grupo de Investigación Traslacional Vascular y Renal, Instituto de Investigación Biomédica IRBlleida, 25198 Lleida, Spain; 15 Department of Nephrology, Hospital Universitario Dr Peset, Universitat de València Fisabio, 46017 Valencia, Spain; 16 Department of Nephrology, University Hospital Clínico, INCLIVA, Valencia University, 46010 Valencia, Spain

**Keywords:** chronic kidney disease, CKD-MBD, calcitriol, vitamin D, calcidiol, secondary hyperparathyroidism, osteoporosis, skeletal fragility

## Abstract

Chronic kidney disease (CKD) is a highly prevalent condition worldwide in which the kidneys lose many abilities, such as the regulation of vitamin D (VD) metabolism. Moreover, people with CKD are at a higher risk of multifactorial VD deficiency, which has been extensively associated with poor outcomes, including bone disease, cardiovascular disease, and higher mortality. Evidence is abundant in terms of the association of negative outcomes with low levels of VD, but recent studies have lowered previous high expectations regarding the beneficial effects of VD supplementation in the general population. Although controversies still exist, the diagnosis and treatment of VD have not been excluded from nephrology guidelines, and much data still supports VD supplementation in CKD patients. In this narrative review, we briefly summarize evolving controversies and useful clinical approaches, underscoring that the adverse effects of VD derivatives must be balanced against the need for effective prevention of progressive and severe secondary hyperparathyroidism. Guidelines vary, but there seems to be general agreement that VD deficiency should be avoided in CKD patients, and it is likely that one should not wait until severe SHPT is present before cautiously starting VD derivatives. Furthermore, it is emphasized that the goal should not be the complete normalization of parathyroid hormone (PTH) levels. New developments may help us to better define optimal VD and PTH at different CKD stages, but large trials are still needed to confirm that VD and precise control of these and other CKD-MBD biomarkers are unequivocally related to improved hard outcomes in this population.

## 1. Introduction

Chronic kidney disease (CKD) is a highly prevalent condition worldwide in which the kidneys are functionally and/or structurally damaged [1,2]. As a result, the kidneys lose their ability to properly excrete waste products and perform certain specific endocrine functions [3]. For example, the kidneys are known to play a crucial role in regulating vitamin D (VD) metabolism by converting VD into its active form [1,25-dihydroxy-VD or calcitriol (CTR)] [3,4]. In people with CKD, this ability is impaired, not only because of the loss of functional kidney tissue as CKD progresses but also because of the important role of the multifactorial and early increase in fibroblast growth factor-23 (FGF23) [5,6]. FGF23 is a bone-derived hormone whose main target organ is the kidney, where it suppresses the transcription of the key activation enzyme, 1α-hydroxylase (CYP27B1), and activates the transcription of the key degradation enzyme, 24-hydroxylase (CYP24A1), in the proximal renal tubules [7,8], thereby leading to reduced availability of CTR. Moreover, the circulating concentration of CTR is a positive regulator of FGF23 secretion in bone, creating a feedback loop between kidney and bone. The intracellular signaling cascades downstream of the FGF receptors that regulate the transcription of these hydroxylases in the proximal renal tubules remain to be elucidated [7], as well as the effects of calcium on FGF23 metabolism [9]. It is also important to consider the early reduction of the important FGF23 cofactor klotho in CKD [10,11]. In the presence of klotho, the FGF23 protein gains bioactivity to influence phosphate (P) homeostasis and VD metabolism [12,13]. Among many other effects [13], independently of CKD itself [14], both increased levels of FGF23 and decreased levels of klotho have been clearly associated with mortality [15,16,17] and survival [18,19].

It is now known that VD deficiency [as defined by serum 25-hydroxy-VD (calcidiol) levels] is very common in the general population worldwide [20,21], and studies have shown that people with CKD are at a higher risk of multifactorial VD deficiency due to dietary restrictions and reduced sunlight exposure, among many other factors [22,23]. VD deficiency has been extensively associated with poor outcomes, including bone disease, cardiovascular disease, and higher mortality [24,25,26]. Indeed, a plethora of pleiotropic effects have been associated with VD, which is partly explained by the fact that extra-renal organs have the enzymatic capacity to convert calcidiol into CTR [22,27]. There is no doubt that the evidence is abundant in terms of the association of almost all negative outcomes with low levels of VD, above all the information poured from experimental studies [27,28,29,30]. In humans, retrospective cohorts, prospective studies, and even meta-analyses have found this association [28,29,30,31], but the recent VITAL randomized clinical trial (RCT) has significantly lowered the previous high expectations regarding the beneficial effects of VD supplementation in the general population [32], and RCTs in CKD have mostly failed—but not all—in their primary objectives [31,33,34,35].

Although controversies still exist [30,35,36,37,38], diagnosis and treatment of VD deficiency [with either nutritional (native) or active forms of VD] have not yet been excluded from nephrology practice and guidelines [22,31,39,40], and despite the poor quality of available evidence, much data may still support VD use in populations with VD deficiency or certain special characteristics, such as CKD patients [36,39,40]. Furthermore, when an optimized prediction tool was recently developed using machine-learning techniques, both parathyroid hormone (PTH) and calcidiol were found to be among the seven variables identified as having the best predictive value for 2-year all-cause mortality in patients with CKD G4-G5 [41].

The purpose of this narrative review is to briefly summarize evolving controversies and useful clinical approaches resulting from recent developments in nephrology, taking into account the low levels of evidence that are very common in all fields of nephrology [42].

## 2. CKD-MBD, Vitamin D, Skeletal Fragility, and Osteoporosis

**Figure 1:**
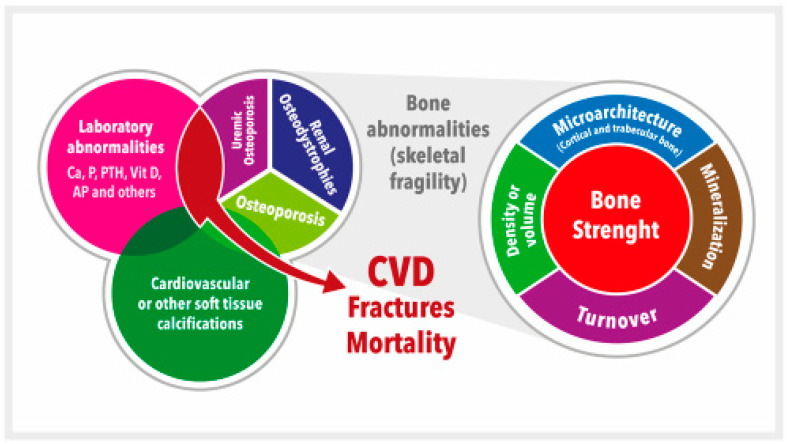
Schematic representation of the chronic kidney disease-mineral and bone disorder (CKD-MBD). CKD-MBD represents a systemic disorder of mineral and bone metabolism due to CKD manifested by either one or a combination of: (a) Laboratory abnormalities [calcium (Ca), phosphate (P), or vitamin D (vitD), among others (i.e., alkaline phosphatase, AP), etc.]; (b) Bone abnormalities in bone turnover, mineralization, volume, etc., ultimately affecting bone strength; and (c) Cardiovascular or other soft tissue calcifications. This figure illustrates the interrelated nature and consequences of CKD-MBD. The area occupied by different concepts is not associated with their relative importance. Adapted from S. Moe et al. (references [43,44]). CVD = Cardiovascular disease.

Since all these complex VD pathophysiology pathways lead to important derangements in CKD, VD is still considered an integral part of the systemic CKD mineral and bone metabolism disorder, now known by the acronym CKD-MBD [39,43,44] (Figure 1). VD plays a vital role in maintaining bone health by promoting intestinal calcium absorption and regulating the activity of osteoblasts and osteoclasts [4,45]. In addition, VD is involved in many other genomic, biochemical, and clinical pathways, with stimulatory or inhibitory effects on the occurrence of morphological and/or functional changes in vital organs, which confer on VD its systemic functions beyond bone [45,46,47]. Specifically, VD deficiency is not merely one of the laboratory abnormalities in need of clinical monitoring but rather is associated with all of the other components (bone disease, vascular calcification, fractures, cardiovascular disease, and mortality) (Figure 1) [39,40,43,44,48]. Moreover, VD deficiency (native or active) has been associated with the development of the well-known CKD-associated secondary hyperparathyroidism (SHPT). In fact, 42%–80% of patients with CKD G3–G4 have SHPT with low serum calcidiol levels and/or other related pathophysiological factors (e.g., increasing P load) [4,39,40,49,50,51,52,53,54]. The incidence of SHPT increases with decreasing renal function, and VD deficiency is more common in patients with CKD than in the general population [39,40,49,55]. Both VD deficiency and SHPT have also been associated with an increased rate of CKD progression, cardiovascular events, and increased mortality [50,56,57,58,59]. This topic is becoming even more relevant with the increasing importance recently given to the diagnosis and potential treatment of skeletal fragility and osteoporosis in CKD patients [39,40,60,61,62,63] (Figure 1). In fact, a very important increase in the risk of fractures has been clearly recognized in patients with CKD, with a multifactorial predisposing factor of muscle weakness and/or risk of falling as contributory factors [39,40,60,61,64,65,66].

## 3. KDIGO Guidelines: From Vitamin D Deficiency to Osteoporosis Treatment

KDIGO (Kidney Disease: Initiative Global Outcomes) 2017 Clinical Practice Guideline Update for the Diagnosis, Evaluation, Prevention, and Treatment of CKD-MBD represented a selective update of the prior guideline published in 2009 [39,67].

Table 1 shows that, in the 2017 update, it was suggested that the potential presence of VD deficiency should be evaluated in patients with CKD G3a-G5 [glomerular filtration rate (GFR) ˂ 60 mL/min/1.73 m^2^, not on dialysis], among other modifiable factors, whenever intact parathyroid hormone (iPTH) levels are progressively rising or persistently above the upper normal limit (UNL) of the assay. VD deficiency is usually corrected with native VD (cholecalciferol, ergocalciferol, or even calcifediol), but dosage (daily, weekly, monthly) and targets are still matters of controversy, with variations among different guidelines [22,39,40,68,69,70,71]. For instance, in early 2011, a committee convened by the Institute of Medicine (IOM) issued a report on the Dietary Reference Intakes (DRI) for calcium and VD (Table 2) [69,70], and in July 2011, the Endocrine Society Task Force published a guideline for the evaluation, treatment, and prevention of VD deficiency [71]. Disagreements concerning the nature of the available data and the resulting conclusions led to some confusion [72], which may be even more evident if one also considers the presence of CKD [73].

Based on bone health, the IOM Recommended Dietary Allowances (RDAs; covering requirements of ≥97.5% of the population) for VD ranged from 600 International Units (IU)/day for ages 1–70 years and 800 IU/day for ages 71 years and older, corresponding to a serum calcidiol level of at least 20 ng/mL (50 nmol/liter). Importantly, the maximum daily intake increased from 2000 to 4000 IU/day [69,70]. In general, experts and most scientific societies concerned with this matter consider VD deficiency to be present at values lower than 20 ng/mL, VD insufficiency at values between 20 and 29 ng/mL, and VD sufficiency at values ≥ 30 ng/mL (which probably should be considered optimal levels of VD) [74,75]. However, additional controversy centers on whether it is necessary to reach 30 ng/mL (or more) to achieve VD effects inside and outside the bone [75,76]. In our experience, at least in non-dialysis CKD patients, a calcidiol level < 20 ng/mL was an independent predictor of death but also progression in CKD patients G3-G5, with no additional benefits when levels at or above 30 ng/mL were reached [24]. However, there are bone biopsy data indicating that at VD levels below 30 ng/mL, the osteoid volume will be higher, and osteomalacia was present in 25.6% of Northern European individuals with such levels [77]. A similar calcidiol threshold was recently reported for optimal bone mineral density (BMD) in the elderly with CKD [78]. The same thresholds were previously associated with SHPT and hip bone loss in a Spanish population of postmenopausal women and men aged 44–93 years old [79]. In fact, PTH could be taken as a potential surrogate marker and an interesting “functional” demonstration of VD deficiency (even disregarding the use of non-standardized VD measurements) [80]. The National Kidney Foundation recommends that people with CKD maintain calcidiol levels between 30 and 60 ng/mL and that adults with CKD consume at least 1000–4000 IU of VD daily [68]. The Italian Association of Clinical Endocrinologists and the Italian Chapter of the American Association of Clinical Endocrinologists recommend that in “categories at risk”, physicians should aim to maintain calcidiol levels above 30 ng/mL [81], and the recent 2020 U.S. KDOQI suggests that patients with CKD G1-G5D should receive supplementation using the same strategies recommended for the general population, recognizing that patients with CKD may require a more aggressive therapeutic plan [74,76]. It is clear, then, that there is conflicting evidence on the optimal levels of VD (with disagreement on both the lower normal limit and an undefined upper limit), not only in the general population but also in CKD patients [76]. Correspondingly, the evidence regarding the safety and efficacy of high-dose VD supplementation in patients with CKD remains discordant [37], and periodic measurement of serum calcium and phosphate should be considered, especially for patients who are using calcium-based phosphate binders and/or VD active analogues [74].

Finally, VD supplementation should also be considered in CKD patients when osteoporosis treatment is started. Importantly, the 2009 KDIGO guidelines suggested that in patients with CKD G3-G5D with evidence of CKD-MBD, BMD testing should not be performed routinely because it was believed that BMD did not predict fracture risk as it did in the general population (evidence level 2B) [67]. Moreover, BMD does not predict the type of “renal osteodystrophy” (ROD). Nevertheless, a very important increase in the risk of fractures was subsequently recognized in patients with CKD [39,40,60,61,64,67], leading to the conclusion that it is “time for action” [60]. In fact, multiple new prospective studies documented that lower BMD (as measured by densitometry) predicts incident fractures in patients with CKD G3a-5D, leading to an important paradigm shift in the 2017 KDIGO guidelines [39]. Thus, the guidelines now suggest that in patients with CKD G3a-G5D with evidence of CKD-MBD and/or risk factors for osteoporosis, BMD testing is appropriate to assess fracture risk if results will impact treatment decisions (evidence level 2B in the opposite direction) [39]. The primary motivation for this revision was the growing experience with osteoporosis medications in patients with CKD, low BMD, and a high risk of fracture and the recognition that the lack of ability to perform a bone biopsy (previously suggested) may not justify withholding antiresorptive therapy [39,67]. Multiple algorithms, society endorsements, and consensus documents followed on the diagnosis and management of osteoporosis in CKD [40,62,63,82,83], including at advanced CKD stages and in dialysis patients [61]. Not only should vitamin D supplements be considered when initiating osteoporosis treatments, but an adequate calcium intake should also be evaluated and reinforced since calcium intake in CKD patients is usually deficient and/or impaired [84,85].

## 4. KDIGO Guidelines: Secondary Hyperparathyroidism and Active Vitamin D

Monitoring iPTH serum levels, beginning at CKD stage G3a or even earlier, has always been recommended in CKD patients [4,39,67,68]. As mentioned above, iPTH has also frequently been associated with hard kidney and/or cardiovascular outcomes, including increased mortality [39,67].

In 2003, the U.S. Kidney Disease Outcomes Quality Initiative (KDOQI) guidelines provided opinion-based ranges for iPTHs that are dependent on CKD stages [68]. Therapy with active oral VD (i.e., CTR) was considered to be indicated in patients with CKD G3–G4 when serum levels of calcidiol were >30 ng/mL and plasma iPTH levels were simply above the suggested target range. The 2009 KDIGO guidelines suggested that CTR or VD analogs (e.g., paricalcitol) could be used in non-dialysis patients in whom serum iPTH is progressively rising and remains persistently above the UNL for the assay despite correction of modifiable factors (evidence level 2C) (Table 1). The same approach was suggested in dialysis patients with elevated or rising iPTH to lower PTH levels towards the suggested goals (2X-9X the UNL or increasing trends in between those values) for CKD-G5D patients (using CTR or VD analogs alone or in combination with calcimimetics). However, in the 2017 KDIGO guidelines, an important change was introduced in that it was stated that it is reasonable to reserve the use of CTR and VD analogs for patients with CKD G4-G5 (GFR ˂30 mL/min/1.73 m^2^, not on dialysis) with severe and progressive SHPT [39] (Table 1). Failure to undoubtedly demonstrate improvements in hard end-points and the increased risk of hypercalcemia (P overload could also have been considered) were drivers for this statement resulting from the PRIMO and OPERA studies [33,34,39,57]. Hypercalcemia can be associated with faster progression of cardiovascular calcification, among other complications, including worsening renal function. Additionally, some patients with CKD are at a higher risk of developing kidney stones, and high doses of VD may increase this risk. However, this important change towards the restriction of active forms of VD, a frequent nephrology practice for many decades [4,68], was not graded. In fact, a clear-cut definition of “severe and progressive SHPT” was not provided, and a controversial discussion preceded the consensus statement [39]. In fact, the primary aim of the PRIMO and OPERA studies was not the biochemical control of SHPT but the potential prevention of the development of left ventricular hypertrophy [33,34]. Furthermore, in these trials, patients only had moderate SHPT, quite unusually high doses of paricalcitol were used, and a significant percentage of study participants received calcium-based P binders [57]. These aspects could probably explain the high incidence of hypercalcemic episodes. Moreover, the study design may have led to an undesired “oversuppression” of PTH secretion and to FGF23 overstimulation [86]. Actually, it is well known that FGF23 may induce left ventricular hypertrophy and thereby counterbalance the potential cardiac improvement with VD [87].

Although some international and national nephrology societies have adopted most of the new 2017 KDIGO suggestions in their position statements, others have not done so for various reasons [40,88,89,90,91]. Some consider that future responses to treatments aiming to control iPTH may be compromised by the delay induced by the guideline update [40,57,59]. Untreated SHPT results in progressively increasing iPTH levels, as observed in RCTs in placebo-treated patients [92,93,94], and increasing parathyroid hyperplasia may reduce sensitivity to calcium and VD regulation [95]. It has been shown that increased iPTH before dialysis inception predicts a higher PTH level 9–12 months later and greater use of anti-parathyroid treatments [96]. Moreover, recent data from a German study show that patients with CKD G3-G4 and incident SHPT of renal origin present with significantly higher all-cause healthcare resource utilization and costs and increased CKD progression as compared with patients without SHPT [97,98].

Interestingly, independent effects and interactions of SHPT, hyperphosphatemia, and hypercalcemia with respect to several outcomes were recently analyzed in the Spanish NEFRONA cohort, which included low-risk CKD G3–G5 patients [99]. In this study, SHPT and hyperphosphatemia (as defined by the old KDOQI guideline targets) and higher iPTH and/or P levels were independently associated with an increased risk of both CKD progression and/or cardiovascular events (a trend for SHPT in the fully adjusted model) [99]. These results offer support for the claim that iPTH levels higher than those specified by the KDOQI for non-dialysis CKD patients are indeed associated with clinically significant hard outcomes [59,99]. The results also underline the need to better define cut-off targets for safe upper PTH levels in non-dialysis patients and to consider whether the KDIGO proposal that active VD analogs should be reserved for severe SHPT is exceedingly cautious [57,59]. It should also be considered that optimal PTH targets are not really known, especially for non-dialysis patients, and that they may be quite different depending on whether bone, renal, or cardiovascular parameters are considered [39,40,57]. In any case, it cannot be forgotten that a certain degree of SHPT may represent an adaptive clinical response, and, accordingly, some recent clinical guidelines underline that clinicians should neither wait until severe SHPT is present nor aim to completely normalize iPTH levels [40,57]. The presence of bone hyporesponsiveness to PTH in CKD [52,100] and the potentially beneficial phosphaturic properties of PTH at least partially explain this last recommendation. On the other hand, PTH is recognized as a uremic toxin [101] and has been associated with many untoward effects and undesirable kidney, cardiovascular, and survival outcomes in observational studies (recently, these effects have even included dementia) [57,102,103]. Moreover, PTH is a recognized inducer of FGF23 transcription in bone cells [104]. Overall, not only should insufficient SHPT control be avoided, excessive suppression of iPTH is undesirable if long-term outcomes are to be improved [40,57]. In fact, low iPTH levels have also been associated with undesirable outcomes, although it is not clear whether the potential development of adynamic (low turnover) bone disease per se or the conditions leading to suppression of bone turnover (chronic inflammation, oxidative stress, malnutrition, diabetes, etc.) are the real cause of the worse prognosis [105,106].

In summary, although SHPT is at the core of classical nephrology, it is clear that there is still no homogeneous approach to the management of VD deficiency and/or SHPT, especially in non-dialysis CKD patients [39,40,57,107,108]. The adverse effects associated with increasing calcium and/or P levels with VD derivatives must be balanced against their potential pleiotropic beneficial effects in CKD patients and the need for effective prevention of progressive and severe SHPT and parathyroid gland autonomy. Guidelines may vary, but there seems to be general agreement that VD deficiency should be avoided, and it is likely that one should not wait until severe SHPT is present before cautiously starting active VD derivatives. Furthermore, the goal should not be complete normalization of iPTH levels. New developments, such as extended-release formulations [109,110] (which correct both vitamin D deficiency and are more effective than native in decreasing iPTH levels) and new analogs, biomarkers, molecular targets, and even renal pathologies [111,112,113,114,115,116,117], may then help us better define optimal VD and iPTH levels or the best formulation at different CKD stages [92,107,109], thereby directing us towards an improved, personalized medicine. It is possible that the approaches that we took to correct VD deficiency are at least partially wrong and that current interventions with native and/or active VD were not properly targeted at more effective goals. This new era of nephrology, in which it is proposed that we return to basics and to a holistic view focusing in particular on the early stages of CKD, is the ideal scenario for the procurement of more evidence in an area of vital importance for cardiovascular health, including the kidney-heart-bone interaction [118,119]. In any case, we cannot sit back, and it is to be emphasized that large RCTs are still needed to confirm that VD and precise control of these and other CKD-MBD biomarkers are directly and unequivocally related to improved hard outcomes in CKD patients.

## Figures and Tables

**Table 1:** Comparison of KDIGO guidelines 2009/2017 on treatment of abnormal PTH levels in non-dialysis patients. Adapted from reference [39].

	KDIGO 2009	KDIGO 2017
Guideline 4.2.1	In patients with CKD G3a-G5 not on dialysis, the optimal PTH level is not known. However, we suggest that patients with levels of intact PTH above the UNL of the assay be first evaluated for hyperphosphatemia, hypocalcemia, and vitamin D deficiency. *(Evidence Level 2C)*	In patients with CKD G3a-G5 not on dialysis, the optimal PTH level is not known. However, we suggest that patients with levels of intact PTH progressively rising or persistently above the UNL for the assay be evaluated for modifiable factors, including hyperphosphatemia, hypocalcemia, high phosphate intake, and vitamin D deficiency. *(Evidence Level 2C)*
Guideline 4.2.2	In patients with CKD G3a-G5 not on dialysis, in whom serum PTH is progressively rising and remains persistently above the UNL for the assay despite correction of modifiable factors, we suggest treatment with calcitriol or vitamin D analogs. *(Evidence Level 2C)*	In adult patients with CKD G3a-G5 not on dialysis, we suggest that calcitriol and vitamin D analogs not be routinely used. *(Evidence Level 2C).* It is reasonable to reserve the use of calcitriol and vitamin D analogs for patients with CKD G4-G5 with severe and progressive hyperparathyroidism. *(Not Graded)*

**Table 2:** Dietary Reference Intakes for Vitamin D (Institute of Medicine). Adapted from references [69,70]. The upper level intake for calcium has not been included since it is much greater than the upper limit considered safe in CKD patients (1000–1200 mg/day).

	Estimated Average Requirement (EAR)	Recommended Dietary Allowance (RDA)	Upper Level Intake
19–70 y/o	*400 IU/day* The EAR for calcium varies from 800 mg/day (19–50 y/o females and 19–70 y/o males) to 1000 mg/day (51–70 y/o females)	*600 IU/day* The RDA for calcium varies from 1000 mg/day (19–50 y/o females and 19–70 y/o males) to 1200 mg/day (51–70 y/o females)	*4000 IU/day*
>70 y/o	*400 IU/day* The EAR for calcium is 1000 mg/day	*800 IU/day* The RDA for calcium is 1200 mg/day	*4000 IU/day*

y/o = years old; IU = international units.
